# INTERCEPT-AD, a phase 1 study of intravenous sabirnetug in participants with mild cognitive impairment or mild dementia due to Alzheimer's disease

**DOI:** 10.1016/j.tjpad.2024.100005

**Published:** 2025-01-01

**Authors:** Eric Siemers, Todd Feaster, Gopalan Sethuraman, Karen Sundell, Vladimir Skljarevski, Erika N. Cline, Hao Zhang, Jasna Jerecic, Lawrence S. Honig, Stephen Salloway, Reisa Sperling, Mirjam N. Trame, Michael G. Dodds, Kimball Johnson

**Affiliations:** a Acumen Pharmaceuticals, Newton, MA, USA; b Columbia University Irving Medical Center, New York, NY, USA; c Butler Hospital and Brown University, Providence, RI, USA; d Harvard Medical School, Boston, MA, USA; e Certara USA, Inc., Princeton NJ, USA; f CenExel iResearch, Atlanta, GA, USA

**Keywords:** Alzheimer's disease, ACU193, Sabirnetug, AΒ oligomers, Target engagement

## Abstract

**Background:**

Soluble species of multimeric amyloid-beta including globular amyloid-beta oligomers (AβOs) and linear amyloid-beta protofibrils are toxic to neurons. Sabirnetug (ACU193) is a humanized monoclonal antibody, raised against globular species of soluble AβO, that has over 650-fold greater binding affinity for AβOs over monomers and appears to have relatively little binding to amyloid plaque.

**Objectives:**

To assess safety, pharmacokinetics, and exploratory measures including target engagement, biomarker effects, and clinical efficacy of sabirnetug in participants with early symptomatic Alzheimer's disease (AD; defined as mild cognitive impairment and mild dementia due to AD).

**Design:**

Randomized, double-blind, placebo-controlled, ascending dose first-in-human phase 1 study.

**Setting:**

Fifteen study centers in the United States.

**Participants:**

Sixty-five participants with early symptomatic AD.

**Intervention:**

Participants received one infusion of sabirnetug 2 mg/kg, 10 mg/kg, 25 mg/kg, 60 mg/kg, or placebo (Part A) or three infusions of sabirnetug 10 mg/kg, 25 mg/kg, 60 mg/kg, or placebo (Part B).

**Measurements:**

Safety, tolerability, serum pharmacokinetics, and central target engagement of single and multiple doses of sabirnetug, cerebrospinal fluid (CSF) concentrations of sabirnetug, and amyloid plaque load, as determined by positron emission tomography.

**Results:**

Sabirnetug was generally well tolerated. A larger percentage of participants receiving sabirnetug (56.3%) versus placebo (42.9%) had at least one treatment emergent adverse event, with approximately 29% in each group considered related to study drug. Most events were mild-to-moderate in severity. Of 48 participants given sabirnetug, five developed amyloid related imaging abnormalities – edema/effusion, including one instance that was mildly symptomatic in a participant who had received one dose sabirnetug 60 mg/kg. Notably, none of the six *apolipoprotein E Ɛ4* homozygotes who received sabirnetug developed amyloid related imaging abnormalities – edema/effusion or – hemorrhage/hemosiderin deposition. Infusion reactions, such as rash, pain, or erythema, were not frequent (6.3% for sabirnetug versus 0.0% for placebo). Sabirnetug exposure was dose proportional in both serum and CSF. Target engagement, defined as drug bound to AβOs in CSF, was shown to be dose and exposure dependent. Over three months, approximately 25% and 20% reduction in amyloid plaques, respectively, were observed in participants receiving three infusions of sabirnetug 60 mg/kg every four weeks and 25 mg/kg every two weeks.

**Conclusions:**

The Phase 1 INTERCEPT-AD study provided safety, tolerability, dosing, and target engagement data that supported the design of the ongoing ALTITUDE-AD study (NCT06335173).

## 1 Introduction

Alzheimer's disease (AD) is an increasing public health issue. Post-COVID, it is the seventh leading cause of death in the United States (US) and, with an aging population, the cost of care for AD in the US alone could approach $1 trillion by 2050 [1]. First described by Alois Alzheimer in 1906, AD is marked by the pathological presence of extracellular amyloid plaques and intracellular hyperphosphorylated tau tangles. Following the emergence of the “amyloid hypothesis” [[2], [3], [4]], the amyloid-beta (Aβ) pathway [5] has been targeted in attempts to find disease-modifying treatments for AD.

Two main strategies to develop disease-modifying therapies related to the amyloid hypothesis involved decreasing the production of Aβ from the amyloid precursor protein (APP) and clearing Aβ from the brain immunologically. Two proteolytic activities lead to the production of Aβ from the longer transmembrane APP protein: the β-site APP-cleaving enzyme (BACE) and gamma secretase. Beta and gamma secretase inhibitors did not slow disease progression in clinical trials and variably showed evidence of slight worsening, likely reversible, of cognition [[6], [7], [8], [9], [10]]. Attempts to lower Aβ levels with monoclonal antibodies directed toward soluble Aβ (monomers) with solanezumab and crenezumab resulted in a trend for slowing disease progression for solanezumab and no effect for crenezumab in patients with mild dementia due to AD [[11], [12], [13], [14]]. A study of higher solanezumab doses in patients with preclinical AD showed a reduced rate of amyloid accumulation on positron emission tomography (PET) but no positive clinical effects [15].

Efforts to target deposited amyloid plaques have produced varying degrees of amyloid clearance, with corresponding degrees of clinical benefit. Bapineuzumab was the first monoclonal immunoglobulin G (IgG) antibody to target amyloid plaques, but modest reductions in plaque load were not accompanied by slowed clinical progression of disease [16]. Amyloid related imaging abnormalities – edema/effusion (ARIA-E) were first described during these studies [17]. Subsequently, efforts to slow disease progression by reducing plaque load have shown clinical benefits only for those agents showing marked reductions: aducanumab, lecanemab, and donanemab. A Phase 3 trial for aducanumab (EMERGE) resulted in significant clinical benefit, but a second study (ENGAGE) had less plaque reduction and did not result in clinical benefit [18]. Phase 3 studies for gantenerumab resulted in relatively modest reductions in amyloid plaque load and some improvement in AD biomarkers, but no statistically significant effect on clinical progression [19]. Studies for lecanemab, targeting Aβ protofibrils, and donanemab, targeting pyroglutamylated Aβ primarily localized in amyloid plaques, showed robust reduction of plaques in Phase 2 and Phase 3 studies [[20], [21], [22], [23]] with statistically significant slowing of clinical progression. Taken together, the results show an association of marked reduction in amyloid plaques and clinical efficacy. These trials of monoclonal antibodies successfully lowering amyloid plaque on PET have all shown ARIA-E.

Over the past 25 years, emerging evidence has indicated that while Aβ monomers and amyloid plaques may not be directly toxic to neurons, soluble species of Aβ including globular Aβ oligomers (AβOs) and linear Aβ protofibrils can impair synaptic function and are toxic to neurons [4,[24], [25], [26], [27], [28]]. Sabirnetug (ACU193) is a humanized, affinity-matured, IgG2 subclass monoclonal antibody with reduced effector function that was raised against globular species of AβO [24]. Sabirnetug has at least a 650-fold greater binding affinity for AβOs than for Aβ monomers and shows limited binding to amyloid plaques [24].

This report details clinical safety (including ARIA-E), pharmacokinetics, and several exploratory pharmacodynamic measures, including central target engagement (sabirnetug bound to AβO), from the INTERCEPT-AD Phase 1 study of sabirnetug in participants with early symptomatic AD (mild cognitive impairment [MCI] or mild dementia due to AD). Detailed analyses of CSF and plasma biomarkers of AD pathology will be reported separately.

## 2 Methods

This randomized, double-blind, placebo-controlled, first-in-human study in participants with early symptomatic AD (INTERCEPT-AD; NCT04931459) was conducted from June 23, 2021 to June 12, 2023.

### 2.1 Study objectives

The primary objective of the INTECEPT-AD study was to determine the safety and tolerability of single and multiple intravenous (IV) doses of sabirnetug. The secondary objective was to estimate serum pharmacokinetics of sabirnetug. The exploratory objectives included assessing the following: CSF sabirnetug concentrations and target engagement, changes in amyloid plaque load as determined by PET imaging, CSF and blood-based concentrations of biomarkers associated with AD, and cognitive assessments.

### 2.2 Ethical considerations

This study took place at 15 study centers in the United States and was conducted in accordance with International Council for Harmonisation (ICH) E6 Good Clinical Practice, the Declaration of Helsinki, and all other applicable regulatory requirements. The protocol and informed consent form was approved by ethics committees at all study sites. Written informed consent for study participation and for *apolipoprotein E* (*APOE*) genotyping was provided by all participants or their legally-authorized representatives.

### 2.3 Study design

**Fig. 1:**
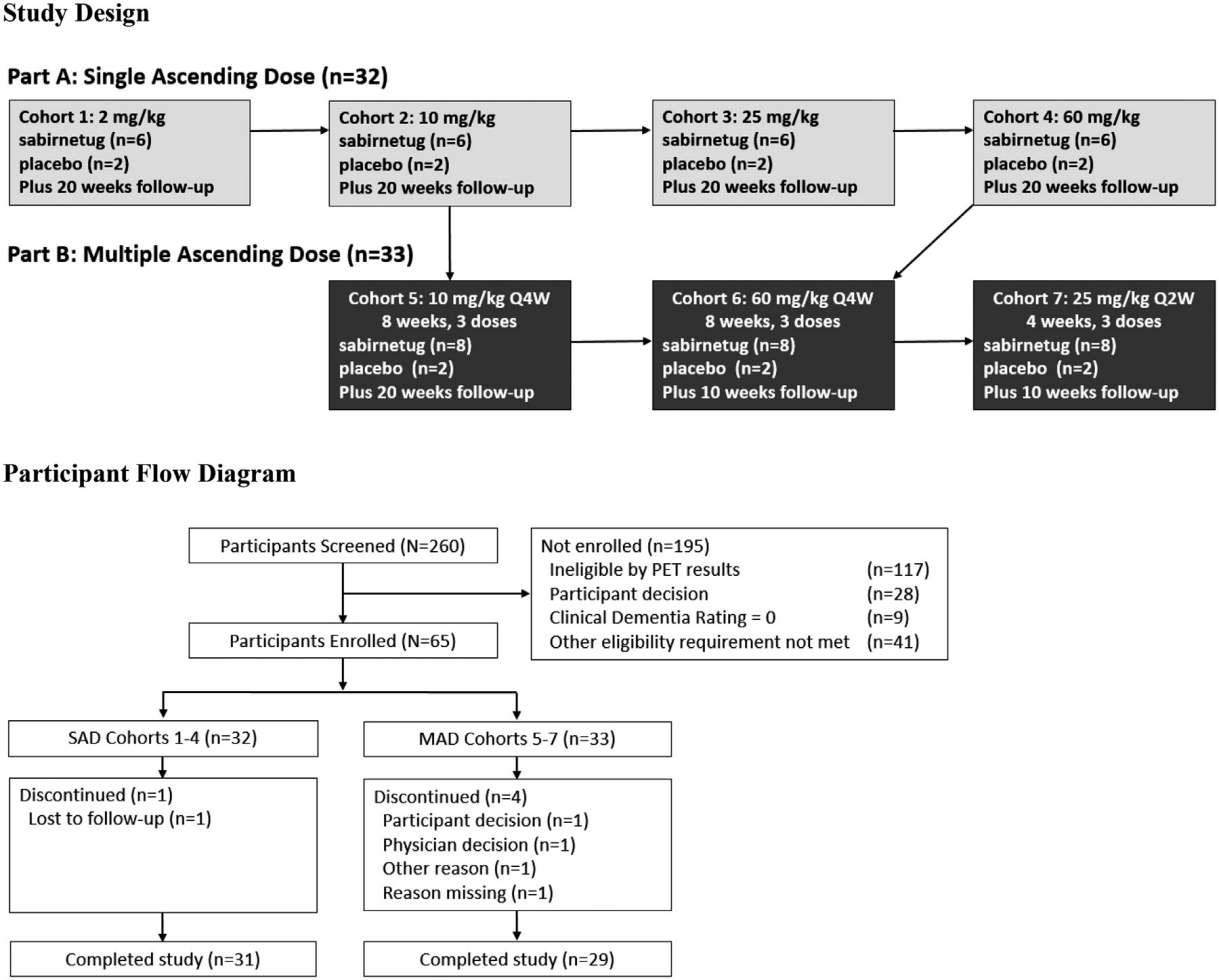
Study Design and Participant Flow Diagram. MAD, multiple ascending dose (study Part B); PET, positron emission tomography; Q2W, once every two weeks; Q4W, once every four weeks; SAD, single ascending dose (study Part A).

INTERCEPT-AD consisted of two parts, A (single ascending dose, SAD) and B (multiple ascending dose, MAD) (Fig. 1). In Part A, four cohorts of participants received single IV infusions of sabirnetug or placebo. In Part B, three cohorts of participants received three infusions each of sabirnetug or placebo. Participants enrolled in Part A were not eligible for enrollment in Part B. The solution used to dilute sabirnetug for infusion was also used as the placebo. Placebo and sabirnetug were both clear, colorless to yellow liquids, identical in appearance.

### 2.4 Design of study Part A

Part A consisted of a seven-week screening period, a double-blind single infusion of study drug, and a 20-week follow-up period. Participants were randomized in a 6:2 ratio to sabirnetug or placebo into one of four cohorts. Participants randomized to sabirnetug in cohorts 1, 2, 3, or 4 received a single IV infusion of 2 mg/kg, 10 mg/kg, 25 mg/kg, or 60 mg/kg sabirnetug, respectively. Participants in the placebo groups received a single IV infusion of placebo. Participants were monitored as inpatients for at least 48 h after study drug administration.

Multiple blinded safety reviews were conducted throughout the trial. Within each cohort, a sentinel dosing scheme was used whereby one of the first two participants to receive the study drug was randomized to sabirnetug and one was randomized to placebo. A blinded safety review was then conducted at least 48 h after dosing of these two participants and before any other participants in the cohort received the study drug. Blinded safety reviews at least one week after completion of cohorts 1 and 2, respectively, were conducted prior to initiation of cohorts 2 and 3. Another blinded safety review, conducted at least one week after completion of cohort 3 and including aggregated preliminary pharmacokinetic data from cohort 2, was required before participants in cohort 4 initiated study drug. An independent Data Monitoring Committee reviewed unblinded safety data twice in study Part A, after completion of dosing in cohort 2 and in cohort 3.

### 2.5 Design of study Part B

Part B consisted of a seven-week screening period and a double-blind treatment period, with participants randomized in an 8:2 ratio to sabirnetug or placebo into one of three cohorts (5, 6, or 7). Participants randomized to sabirnetug in cohorts 5 or 6, respectively, received three IV infusions of sabirnetug 10 mg/kg or 60 mg/kg administered once every four weeks (Q4W). Participants in these two cohorts who were randomized to placebo received three IV infusions of placebo administered Q4W. Participants in cohort 7 received three IV infusions of study drug administered once every two weeks (Q2W), either sabirnetug 25 mg/kg or placebo. Participants were monitored for four hours following the first IV infusion and for two hours following subsequent IV infusions.

Multiple blinded safety reviews were also conducted in Part B. Blinded safety reviews were completed at least one week after completion of SAD cohorts 2 and 4, respectively, before initiation of dosing for MAD cohorts 5 and 6. Before initiation of dosing for cohort 7, a blinded safety review was conducted at least one week after completion of SAD cohorts 3 and 4, which also included aggregated preliminary pharmacokinetic data from cohort 3. Participants in cohorts 5, 6, and 7 were followed for twenty, ten, and ten weeks, respectively, following final study drug infusion. An independent Data Monitoring Committee reviewed unblinded safety data twice in study Part B, after completion of dosing in cohort 4 and midway through dosing in cohort 6.

### 2.6 Participants

Eligible participants were men and women aged 55 to 90 years who met the National Institute on Aging – Alzheimer's Association criteria for MCI or mild dementia due to AD, had a Global Clinical Dementia Rating score (CDR-GS) of 0.5 or 1.0, Mini-Mental State Examination (MMSE) scores between 18 and 30 (inclusive), and positive amyloid PET scans. Amyloid PET scans were considered positive if the composite standard uptake value ratios (SUVr) score was >1.2 (>26.2 Centiloids) and negative if the composite SUVr score was <1.0 (<−8.7 Centiloids). Scans with SUVr values between 1.0 and 1.2 were submitted for visual read. Participants were required to weigh between 41 kg and 113 kg at screening, and those treated with cholinesterase inhibitors or memantine for AD-related symptoms were required to have stable doses of these treatments for at least four weeks prior to baseline and throughout the duration of the study.

Individuals were excluded if they had received any investigational biological drug or any approved treatments that targeted amyloid plaques in the brain less than one year prior to baseline or any investigational small molecule drug less than six months prior to baseline or if they were receiving or were likely to require oral anticoagulants (antiplatelet drugs were permitted), or if they had a history of significant neurological disease, other than AD. Other exclusion criteria included magnetic resonance imaging (MRI) or computed tomography (CT) imaging of the brain within the previous two years that showed pathology inconsistent with a diagnosis of AD, MRI showing >4 amyloid-related imaging abnormalities – hemorrhage/hemosiderin deposition (ARIA-H), presence of any ARIA-E, or any superficial siderosis; history or presence of clinically significant abnormal electrocardiogram (ECG) or an ECG with QT interval corrected using Fridericia's formula (QTcF) >470 msec for women or >450 msec for men; or clinically significant ongoing cardiac or cerebrovascular disease; or current symptoms of major depressive disorder or any current primary psychiatric diagnosis likely to confound interpretation of drug effect, affect cognitive assessments, or affect the participant´s ability to complete the study. Individuals were also excluded if they were considered a suicide risk as assessed by question 5 of the Columbia-Suicide Severity Rating Scale (Active Suicidal Ideation with Specific Plan and Intent) in the last six months, or suicide attempt within the last six months, or investigator opinion. Finally, they were excluded if they had a history of multiple concussions, significant head trauma, or objective change in neuropsychological function within the last five years; or had any history of human immunodeficiency virus or a history of alcohol or drug abuse/dependence within the last five years.

### 2.7 Endpoints and assessments

Safety assessments included adverse events, standard safety panel laboratory tests, vital signs, ECGs, immunogenicity testing for anti-drug antibodies (ADA), MRI scans for ARIA-H and ARIA-E, and the children's version of the Columbia Suicide Severity Rating Scale (C-SSRS). Immunogenicity of sabirnetug was assessed using a sequential approach designed to screen serum for ADA activity, confirm the sabirnetug-specificity of ADA activity, and quantify confirmed activity by titer determination. Methods were developed, validated, and applied to test specimens in a blinded manner to pre- and post-treatment clinical trial specimens (LabCorp, Indianapolis, IN).

Cognitive assessments included change from baseline in scores on the Alzheimer Disease Assessment Scale – Cognitive Subscale, 13-item version (ADAS-Cog13), computerized cognitive testing battery (CCTB), MMSE, Clinical Dementia Rating – Sum of Boxes and Global Score (CDR-SB, CDR-GS), Alzheimer's Disease Cooperative Study-Activities of Daily Living (ADCS-ADL) and Neuropsychiatric Inventory, 10 item version (NPI). For cohorts 1–4 in Part A, all cognitive assessments were collected at baseline and day 21 except for CCTB which was collected at baseline and days 2, 3, 4, 7, and 21. During Part B, preliminary pharmacokinetic data indicated the half-life of sabirnetug in humans was shorter than originally estimated based studies in nonhuman primates. Therefore, the time to Visit 6 and the end-of-study visit was shortened for cohorts 6 and 7, resulting in earlier cognitive assessments for those cohorts. Cognitive assessments for cohort 5 were collected at baseline and days 70 and 196 (14 and 140 days after last dose) except for NPI (collected at baseline and day 70) and CCTB (collected at baseline and days 7, 28, 56, 70, and 196). Cognitive assessments for cohort 6 were collected at baseline and days 63 and 126 (7 and 70 days after last dose) except for NPI (collected at baseline and day 63) and CCTB (collected at baseline and days 7, 28, 56, 63, and 126). Cognitive assessments for cohort 7 were collected at baseline and days 35 and 98 (7 and 70 days after last dose) except for NPI (collected at baseline and day 35) and CCTB (collected at baseline and days 7, 28, 35, and 98). The day 196, 126, and 98 collections (140, 70, and 70 days after last dose) for cohorts 5, 6, and 7 respectively, were considered to be after drug washout.

### 2.8 Pharmacokinetics and pharmacodynamics

Serum pharmacokinetic parameters estimated for Parts A and B included the area under the serum concentration-time curve (AUC) from time 0 to the time of last measurable concentration (AUC_t_), the maximum observed serum concentration, (C_max_[observed]), time to reach C_max_(observed) (T_max_[observed]), AUC from time 0 to infinity (AUC_∞_), elimination half-life (T_1/2_), clearance (CL), apparent volume of distribution at terminal phase (V_z_), and accumulation as assessed by the ratio of C_max_ and AUC_∞_ on day 56 relative to day 0 for cohorts 5 and 6 or by the ratio of day 28 relative to day 0 for cohort 7. Accumulation ratios were determined for MAD cohorts 5, 6, and 7 by comparing AUC and C_max_ values following the first and third doses.

CSF sampling by lumbar puncture was performed at baseline and day 21 (SAD, cohorts 1–4), and on days 70, 63, and 35 (14, 7, and 7 days after last dose) for MAD cohort 5, 6 and 7, respectively. CSF samples were used to measure concentrations of sabirnetug, target engagement, and multiple biomarkers of AD pathology.

### 2.9 Target engagement assay

The target engagement assay used a sabirnetug-specific anti-idiotype capture antibody and an AβO selective detection antibody within the ultrasensitive MSD S-Plex Turbo immunoassay platform (Meso Scale Diagnostics, Rockville, MD), a specific and selective assay for the sabirnetug-AβO complex in human CSF. CSF samples were quantitated relative to a sabirnetug-AβO calibrator and reported as arbitrary units (AU)/mL [30].

The exposure-response relationship between sabirnetug and sabirnetug-AβO complex in CSF was explored using an E_max_ model (E=E_max_*C/(C+EC_50_), where E=target engagement, C=sabirnetug concentration in CSF, E_max_=maximum target engagement, and EC_50_=sabirnetug CSF concentration eliciting half maximal target engagement.

### 2.10 Positron emission tomography

PET imaging with florbetapir was performed at screening and at day 42 for participants in Part A (SAD) and days 70, 63, 70 (14, 7, and 42 days after last dose) for participants in Part B (MAD cohorts 5, 6, and 7), respectively. PET signals were quantified using SUVr values to estimate brain concentrations of amyloid plaque in composites of brain areas expected to be widely affected by AD pathology (frontal, parietal, lateral temporal, sensorimotor, and anterior and posterior cingulate cortices) compared to the cerebellum which is expected to be spared such pathology. SUVr values were converted to the Centiloid scale using the formula Centiloids=(SUVr*174.5415856813) - 183.210370061647.

### 2.11 Statistical analyses

All statistical analyses were tested at α=0.05, two-sided significance level. Descriptive statistics for continuous variables include number of participants, mean, and standard deviation (SD). Descriptive statistics for categorical variables consist of frequency and percentage. No formal statistical analyses were performed on the safety data. All analyses and tabulations were performed using SAS version 9.4.

### 2.12 Sample size

The sample sizes for Parts A and B are consistent with those used for safety, pharmacokinetic, and pharmacodynamic determinations in early-phase clinical development. The 65 participants enrolled in this Phase 1 study in Part A (*N*=32) and Part B (*N*=33) were expected to provide sufficient safety and biomarker information for further development of sabirnetug.

### 2.13 Analysis populations

The intent-to-treat (ITT) population includes all randomized participants. The safety population, which includes all participants who received at least one infusion of study drug, was used for demographic, disposition, and safety summaries. The modified ITT (mITT) population, which includes all randomized participants who received at least one infusion and had at least one post-infusion efficacy or pharmacodynamic evaluation, was used for efficacy and pharmacodynamic summaries and analyses. The pharmacokinetic population, which includes all participants who completed sabirnetug pharmacokinetic sampling and had sufficient concentration data above the limit of quantitation to allow calculation of pharmacokinetic parameters using validated methods, was used for pharmacokinetic summaries and analyses.

## 3 Results

### 3.1 Participants

Of the 260 individuals screened, 65 were deemed eligible and enrolled in the study (75% screen failure rate). Of the 32 participants enrolled in Part A of the study, 24 were randomized to sabirnetug and 8 to placebo. In Part B, 33 participants were randomized to sabirnetug (*N*=25) or placebo (*N*=8), but three Part B participants discontinued prior to dosing (Fig. 1). Slightly more study participants were women (53.8%) than men. Three (4.6%) participants were African American/Black, one (1.5%) was an Alaskan native, and ten (15.4%) were Hispanic (Table 1). Mean (SD) age was 72.3 (7.9) years for participants receiving sabirnetug and 71.3 (7.3) years for those receiving placebo. Baseline characteristics were balanced between participants receiving sabirnetug and placebo, respectively, for mean (SD) body mass index (28.0 [5.4] kg/m^2^ and 28.9 [5.7] kg/m^2^), MMSE score (24.1 [3.7] and 24.8 [3.6]), CDR-GS score (0.6 [0.3] and 0.6 [0.2]), and CDR-SB score (3.6 [1.9] and 3.2 [1.8]). Smaller percentages of sabirnetug-treated participants compared to participants receiving placebo were homozygous for *APOE* ε4 (12.5% versus 14.5%) or heterozygous for *APOE* ε4 (43.8% versus 57.1%). The number in the ITT population was 65 and in the mITT and safety populations was 64.

**Table 1:** Participant Demographics and Baseline Characteristics

Mean (SD) unless otherwise indicated	Part A (Single Ascending Dose)							
	Cohort 1		Cohort 2		Cohort 3		Cohort 4	
	SABI (*N*=6)	PBO (*N*=2)	SABI (*N*=6)	PBO (*N*=2)	SABI (*N*=6)	PBO (*N*=2)	SABI (*N*=6)	PBO (*N*=2)
**Age, years**	76.0 (7.2)	78.0 (2.8)	72.0 (9.5)	80.5 (3.5)	72.7 (8.7)	68.5 (13.4)	71.8 (8.9)	67.5 (12.0)
**Men, n (%)**	4 (66.7)	2 (100.0)	5 (83.3)	1 (50.0)	3 (50.0)	1 (50.0)	2 (33.3)	1 (50.0)
**Race, n (%)** African American/Black Asian Native American/Alaskan White	1 (16.7) 0 0 5 (83.3)	0 0 0 2 (100)	1 (16.7) 0 0 5 (83.3)	0 0 0 2 (100)	1 (16.7) 0 0 5 (83.3)	0 0 0 2 (100)	0 0 0 6 (100)	0 0 0 2 (100)
**Hispanic Ethnicity, n (%)**	2 (33.3)	0	1 (16.7)	0	0	0	2 (33.3)	1 (50)
**MMSE Total Score**	25.5 (3.7)	27.5 (2.1)	22.8 (3.5)	23.5 (5.0)	24.2 (4.5)	23.0 (1.4)	25.5 (2.4)	22.5 (3.5)
**CDR-GS**	0.6 (0.2)	0.5 (0.0)	0.6 (0.2)	0.8 (0.4)	0.8 (0.3)	0.5 (0.0)	0.6 (0.2)	0.5 (0.0)
**CDR-SB**	3.4 (1.9)	4.0 (0.7)	4.0 (1.8)	4.5 (3.5)	3.8 (1.6)	2.0 (0.0)	3.8 (1.2)	3.0 (2.1)
***ApoE*, n (%)** *e2e3* *e2e4* *e3e3* *e3e4* *e4e4*	1 (16.7) 0 3 (50) 2 (33.3) 0	0 0 1 (50) 1 (50) 0	0 0 2 (33.3) 4 (66.7) 0	0 0 0 2 (100) 0	0 1 (16.7) 3 (50) 1 (16.7) 1 (16.7)	0 0 2 (100) 0 0	0 1 (16.7) 2 (33.3) 3 (50) 0	0 0 0 1 (50) 1 (50)
**Prior Anti-Dementia Medications** Donepezil Galantamine Memantine Memantine-donepezil Rivastigmine	2 (33.3) 2 (33.3) 0 1 (16.7) 0 0	0 0 0 0 0 0	2 (33.3) 1 (16.7) 1 (16.7) 1 (16.7) 0 0	1 (50.0) 0 0 1 (50.0) 0 1 (50.0)	3 (50.0) 2 (33.3) 0 2 (33.3) 0 0	1 (50.0) 1 (50.0) 0 1 (50.0) 0 0	4 (66.7) 2 (33.3) 0 3 (50.0) 0 0	1 (50.0) 1 (50.0) 0 0 0 0


Mean (SD) unless otherwise indicated	Part B (Multiple Ascending Dose)						Parts A and B
	Cohort 5		Cohort 6		Cohort 7		All SABI All SABI (*N*=49)§
	SABI (*N*=8)	PBO (*N*=4)*	SABI (*N*=8)	PBO (*N*=2)	SABI (*N*=9)†	PBO (*N*=2)	
**Age, years**	70.6 (8.6)	67.8 (2.6)	72.1 (8.7)	70.5 (6.4)	71.9 (6.3)	70.0 (5.7)	72.3 (7.9)
**Men, n (%)**	4 (50)	3 (75)	3 (37.5)	0	1[11]	0	22 (44.9)
**Race, n (%)** African American/Black Asian Native American/ Alaskan White	0 0 1 (12.5) 7 (87.5)	0 0 0 4 (100)	0 0 0 8 (100)	0 0 0 2 (100)	0 0 0 9 (100)	0 0 0 2 (100)	2 (4.1) 0 1 (2.0) 46 (93.9)
**Hispanic Ethnicity, n (%)**	0	1(25)	2(25)	0	1(11)	0	8 (16.3)
**MMSE Total Score**	23.8 (3.7)	28.5 (2.1)	23.0 (4.4)	23.5 (6.4)	24.3 (4.0)	25.0 (4.2)	24.1 (3.7)
**CDR-GS**	0.8 (0.3)	0.5 (0.0)	0.6 (0.2)	1.0 (0.0)	0.7 (0.5)	0.5 (0.0)	0.6 (0.3)
**CDR-SB**	4.2 (1.8)	1.8 (0.4)	2.9 (1.2)	5.3 (1.1)	3.6 (3.1)	1.8 (0.4)	3.6 (1.9)
***ApoE*, n (%)** *e2e3* *e2e4* *e3e3* *e3e4* *e4e4*	1 (12.5) 0 3 (37.5) 2 (25) 2 (25)	0 0 1 (50) 1 (50) 0	0 0 4 (50) 3 (37.5) 1 (12.5)	0 0 0 1 (50) 1 (50)	0 0 2(25) 4 (50) 2(25)	0 0 0 2 (100) 0	2 (4.2) 2 (4.2) 19 (39.6) 19 (36.6) 6 (12.5)
**Prior Anti-Dementia Medications** Donepezil Galantamine Memantine Memantine-donepezil Rivastigmine	4 (50.0) 4 (50.0) 0 3 (37.5) 0 0	2 (50.0) 1 (25.0) 0 1 (25.0) 0 0	5 (62.5) 2 (25.0) 0 1 (12.5) 1 (12.5) 1 (12.5)	2 (100.0) 1 (50.0) 1 (50.0) 0 0 1 (50.0)	2 (22.2) 2 (22.2) 0 0 0 0	0 0 0 0 0 0	22 (44.9) 15 (30.6) 1 (2.0) 11 (22.4) 1 (2.0) 1 (2.0)

*ApoE, Apolipoprotein E*; CDR-GS, Clinical Dementia Rating – Global Score; CDR-SB, Clinical Dementia Rating – Sum of Boxes; MMSE, Mini-Mental State Examination; PBO, Placebo; SABI, sabirnetug.

*Sample size for MMSE total score, CDR-GS score, CDR-SB score and *ApoE, n* = 2.

†Sample size for MMSE total score, CDR-GS score, CDR-SB score, and *ApoE, n* = 8.

§Sample size for MMSE total score, CDR-GS score, CDR-SB score, and *ApoE, n* = 48.

### 3.2 Participant disposition

All (100%) of the 32 participants enrolled in Part A of the study received a single infusion of study drug and 31 (96.9%) completed the 20-week follow-up period (Fig. 1). One sabirnetug-treated participant in cohort 2 was lost to follow-up after the infusion. In Part B, 29 (85.3%) of the 33 participants received three infusions of study drug and completed the study. Two (8%) participants who received sabirnetug and two (25%) participants who received placebo discontinued before study completion. Among the sabirnetug-treated participants who discontinued early one received a single partial infusion and one received a single full infusion.

### 3.3 Treatment-emergent adverse events

Treatment-emergent adverse events (TEAEs) by cohort are shown in Table 2. In the study overall, most participants (*N*=33 [53.2%]) experienced at least one TEAE. Overall, a larger percentage of participants receiving sabirnetug versus placebo had at least one TEAE (56.3% versus 42.9%) and at least one TEAE considered related to the study drug (29.2% versus 28.6%). Infusion reactions, an adverse event of special interest, were also observed in a greater percentage of sabirnetug-treated participants compared with placebo (6.3% versus 0.0%), and included maculopapular rash, myalgia, arthralgia, and erythema on the face considered likely to be due to hypersensitivity. Investigators considered most TEAEs to be mild-to-moderate in severity. Four sabirnetug-treated participants experienced severe TEAEs (one participant each with fall and syncope, ARIA-E, ovarian fibroma, and pneumonia). Three serious TEAEs occurred in two sabirnetug-treated participants: pneumonia and mental status change in one participant and ovarian fibroma in another. The pneumonia was considered severe. The other serious TEAEs were considered moderate in severity by the investigator. All serious TEAEs were considered not related or unlikely to be related to study drug. No deaths occurred during the study.

**Table 2:** Treatment Emergent Adverse Events Occurring in ≥5% of All Sabirnetug-Treated Participants and in a Greater Percentage of Sabirnetug-Treated Participants than Participants who Received Placebo

**Preferred Term**	**Single Ascending Dose (Part A)**				
	**Cohort 1** **2 mg/kg** **(*N*=6)**	**Cohort 2** **10 mg/kg** **(*N*=6)**	**Cohort 3** **25 mg/kg** **(*N*=6)**	**Cohort 4** **60 mg/kg** **(*N*=6)**	**Pooled Placebo (*N*=8)**
Participants with ≥1 TEAE	4 (66.7)	3 (50.0)	2 (33.3)	4 (66.7)	5 (62.5)
COVID-19	2 (33.3)	0	0	0	0
ARIA-H*	0	0	1 (16.7)	1 (16.7)	1 (12.5)


**Preferred Term**	**Multiple Ascending Dose (Part B)**				
	**Cohort 5** **10 mg/kg Q4W** **(*N*=8)**	**Cohort 6** **60 mg/kg Q4W** **(*N*=8)**	**Pooled Placebo** **Q4W** **(*N*=4)**	**Cohort 7** **25 mg/kg Q2W** **(*N*=8)**	**Placebo** **Q2W** **(*N*=2)**
Participants with ≥1 TEAE	5 (62.5)	5 (62.5)	1 (25.0)	4 (50.0)	0
ARIA-H*	0	1 (12.5)	0	1 (12.5)	0
ARIA-E	1 (12.5)	2 (25.0)	0	1 (12.5)	0
Diarrhea	0	1 (12.5)	0	1 (12.5)	0
Hypersensitivity†	1 (12.5)	0	0	1 (12.5)	0

TEAE, treatment-emergent adverse event; ARIA-E, amyloid related imaging abnormality-edema/effusion; ARIA-H, amyloid related imaging abnormality- hemorrhage/hemosiderin deposition; Q2W, every 2 weeks; Q4W, every 4 weeks.

*All incidences of ARIA-H were microhemorrhages except for one instance of superficial siderosis in cohort 7.

†Hypersensitivity indicated reactions deemed to be related to infusion.

As assessed using the C-SSRS at baseline, eight participants reported having suicidal ideation and/or behavior at some point in their life. Seven of these participants were randomized to sabirnetug and one was randomized to placebo. Three of these eight participants also reported having suicidal ideation during the study: two sabirnetug-treated participants and one participant who received placebo. No participants without a prior history of suicidal ideation experienced suicidal ideation during the study. No participants had suicidal behavior during the study.

Treatment-emergent anti-sabirnetug antibodies were reported at low titers (ranging from 1 to 8) for six sabirnetug-treated participants: one among the SAD cohorts and five among the MAD cohorts. No placebo-treated participants exhibited treatment-emergent immunogenicity.

### 3.4 Amyloid related imaging abnormalities

**Fig. 2:**
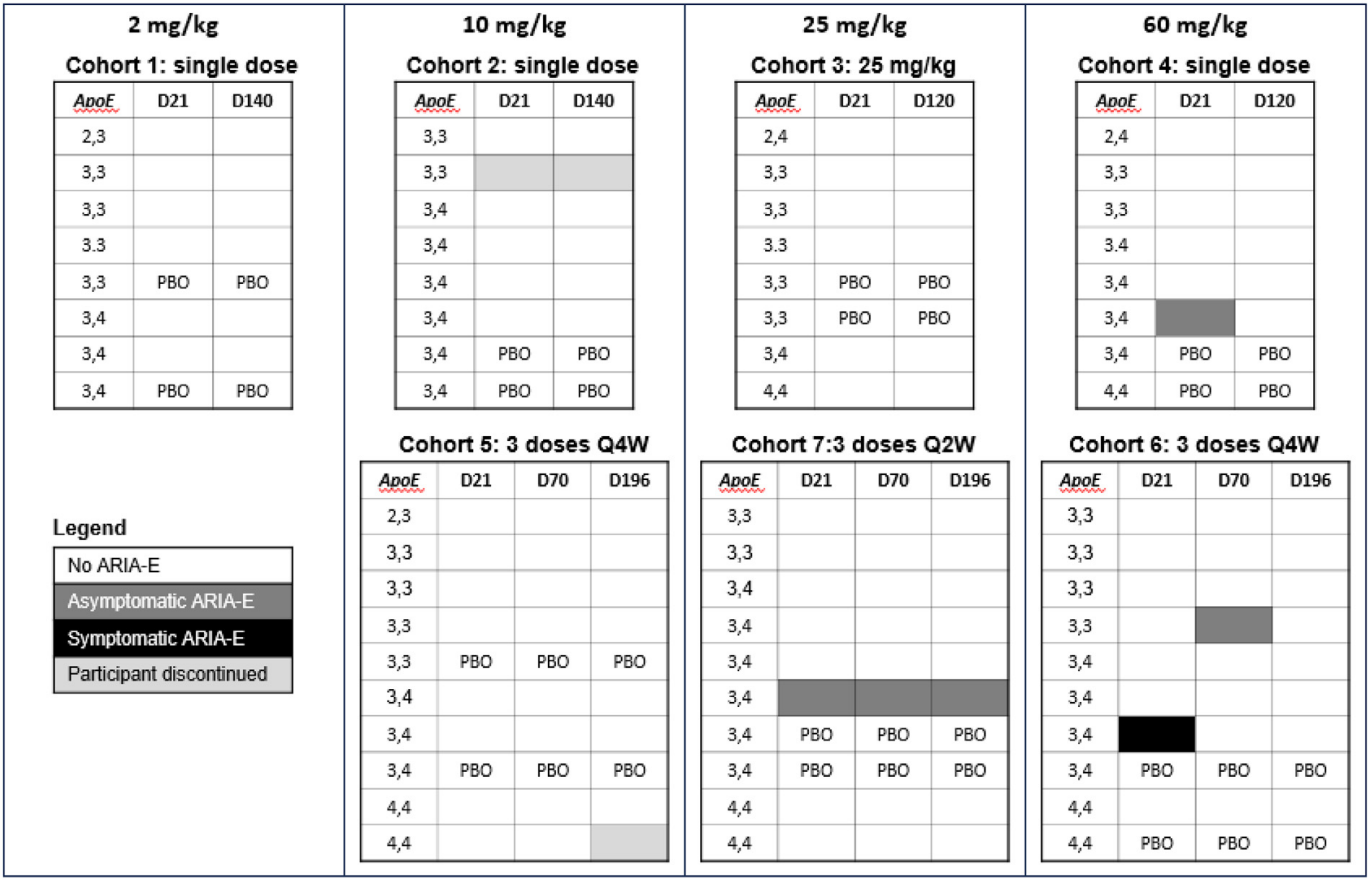
Amyloid Related Imaging Abnormalities – Edema/Effusion. Participants who received placebo are indicated. All other participants received sabirnetug at the dosage shown. *ApoE, apolipoprotein E;* ARIA-E, amyloid related imaging abnormalities – edema/effusion; D, day; PBO, participants who received placebo; Q2W, once every two weeks; Q4W, once every 4 weeks.

The percentage of participants with incident ARIA-E during the study was higher with sabirnetug versus placebo (10.4%, five participants versus 0.0%) (Fig. 2), and all participants who experienced ARIA-E were women. Of the five participants with ARIA-E, one received 60 mg sabirnetug in the SAD portion of the study. The other four were in the MAD portion of the study: one participant who received three administrations of 10 mg/kg sabirnetug Q4W (7% of all participants given 10 mg/kg sabirnetug), one received three administrations of 25 mg/kg sabirnetug (7% of all participants given 25 mg/kg sabirnetug), and two received either one or three doses of 60 mg/kg sabirnetug. Overall, 21% of all participants who received at least one dose of 60 mg/kg experienced ARIA. The only symptomatic participant received a single infusion of 60 mg/kg sabirnetug and experienced mild leg dysfunction which resolved within four weeks as did the finding of ARIA-E in the left frontal region of the brain. Four of five participants who experienced ARIA-E, including the symptomatic participant, were heterozygous for *APOE* ε*4*. The remaining participant was homozygous for *APOE* ε3. None of the six participants who were homozygous for *APOE* ε4 experienced ARIA-E.

The percentage of participants with incident ARIA-H was marginally higher in sabirnetug (8.3%, four participants) versus placebo (7.1%, one participant). Five total ARIA-H cases occurred (four microhemorrhages, one superficial siderosis). Four cases of ARIA-H were in sabirnetug-treated participants, and one occurred in the placebo group. Two cases of ARIA-H occurred with ARIA-E and three were isolated. All participants with new microhemorrhages had had microhemorrhages at baseline, and their number of microhemorrhages increased by one during the study. All participants remained within the limit of four or fewer microhemorrhages allowed by the study entry criteria.

### 3.5 Pharmacokinetics and pharmacodynamics

The pharmacokinetic population (all participants treated with sabirnetug) was 48. For the SAD cohorts 1, 2, 3, and 4 (2, 10, 25, and 60 mg/kg), serum sabirnetug exposure was dose-related and generally dose-proportional as indicated by clearance, dose-normalized C_max_, and AUC (Table 3). In Part A, mean clearance (SD) was cohort 1: 58.93 (12.98) mL/hour, cohort 2: 50.80 (12.08) mL/hour, cohort 3: 50.57 (11.50) mL/hour, cohort 4: 52.54 (10.32) mL/hour and mean C_max_ (SD) was cohort 1: 45 (6.4) µg/mL, cohort 2: 218 (22.5) µg/mL, cohort 3: 618 (72.1) µg/mL, cohort 4: 1,156 (271.9) µg/mL. In Part B, C_max_ following last dose for cohort 5 (10 mg/kg Q4W): 201 (32.5) µg/mL, cohort 6 (60 mg/kg Q4W): 1,271 (465.6) µg/mL, and cohort 7 (25 mg/kg Q2W): 664 (133.1) µg/mL. Serum concentrations peaked near the end of infusion. T_1/2_ and V_z_ appeared to increase with increasing dose; however, these parameters are dependent on the estimation of the terminal slope. At lower doses, serum concentrations dropped below the limit of detection for the assay (0.8 µg/mL) earlier than at higher doses, contributing to the differences in the estimates of the slope.

**Table 3:** Pharmacokinetic Parameters

Variable Mean (SD) unless otherwise stated	Single Ascending Dose (Study Part A)			
	Cohort 1 2 mg/kg (*N*=6)	Cohort 2 10 mg/kg (*N*=6)	Cohort 3 25 mg/kg (*N*=6)	Cohort 4 60 mg/kg (*N*=6)
AUC_inf_, hours*µg/mL	2,867 (855.5)	15,168 (2,740.2)	43,548 (6,432.3)	86,944 (15,709.7)
DN AUC_inf_, hours*µg/mL/mg	18 (4.2)	21 (4.4)	21 (4.8)	20 (3.7)
C_max_, µg/mL	45 (6.4)	218 (22.5)	618 (72.1)	1,156 (271.9)
DN C_max_, µg/mL/mg	0.28 (0.03)	0.29 (0.08)	0.29 (0.04)	0.26 (0.02)
T_max_, hours, median (minimum, maximum)	1.04 (0.98, 6.00)	1.00 (0.23, 2.90)	1.00 (0.92, 1.17)	1.00 (0.90, 5.83)
T_1/2_, hours	76.84 (62.98)	152.8 (56.03)	283.1 (211.5)	391.4 (271.2)
CL, mL/hour	58.93 (12.98)	50.80 (12.08)	50.57 (11.50)	52.54 (10.32)
Vz, L	5.88 (3.32)	11.44 (5.45)	18.21 (10.06)	27.00 (14.77)

Variable Mean (SD)	Multiple Ascending Dose (Study Part B)			
	Cohort 5 10 mg/kg Q4W (*N*=8)	Cohort 6 60 mg/kg Q4W (*N*=8)	Cohort 7 25 mg/kg Q2W (*N*=8)	

First Dose				
AUC_tau_, hours*µg/mL	16,240 (3,213.4)	103,701 (24,797.1)	39,201 (6,165.86)	
C_max_, µg/mL	191 (42.3)	1,454 (538.8)	565 (119.1)	
Last Dose				
AUC_tau_, hours*µg/mg	24,383 (4,557.1)	126,243 (42,114.9)	51,728 (10,237.1)	
C_max_, µg/mL	201 (32.5)	1,271 (465.6)	664 (133.1)	
Dose 3 to Dose 1 AUC_tau_ Accumulation Ratio	1.44 (0.188)	1.25 (0.314)	1.32 (0.220)	
Dose 3 to Dose 1 C_max_ Accumulation Ratio	1.00 (0.069)	0.97 (0.204)	1.18 (0.174)	

AUC_inf_, area under the concentration-time curve from time zero to infinity; AUC_tau_, area under the concentration time curve for the dosing period (tau); CL, clearance; C_max_, maximum serum concentration; DN, dose normalized; Q2W, once every two weeks; Q4W, once every four weeks; T_1/2_, terminal half-life, T_max_, time to reach maximum serum concentration; SD, standard deviation; V_z_, apparent volume of distribution at terminal phase.

Exposure observed with multiple doses was generally dose-proportional and similar to the single dose cohorts. Minimal accumulation with repeated sabirnetug dosing was observed for C_max_ and modest accumulation was observed for AUC over the dosing interval (AUC_tau_).

### 3.6 Concentrations of sabirnetug in cerebrospinal fluid

Concentrations of sabirnetug in CSF were determined using CSF samples collected at day 21 for participants who received a single dose of sabirnetug and at day 70, 63, or 35 (14, 7, and 7 days after last dose) for participants in cohorts 5, 6, and 7, respectively. Concentrations increased with increasing sabirnetug dose. Median concentration (range) of sabirnetug in the CSF were cohort 1: 15.7 (6.7–29.2) ng/mL, cohort 2: 98.0 (36.8–130.7) ng/mL, cohort 3: 169.5 (65.0–334.7) ng/mL, cohort 4: 282.7 (26.1–455.9) ng/mL, cohort 5: 148.9 (5.2–255.9) ng/mL, cohort 6: 1,161.6 (48.1–1,722.2) ng/mL, and cohort 7: 869.8 (419.0–1,474.1) ng/mL.

### 3.7 Percent ratios of mean CSF to serum concentrations of sabirnetug

Sabirnetug concentration measured in CSF was total concentration (bound and unbound) and in serum was free concentration (unbound). Following single dose administration, mean CSF to serum percent ratios at ∼21 days post-dose for sabirnetug ranged from 1.71% to 3.25%. Following multiple dose administrations, mean CSF to serum percent ratios for sabirnetug Q4W ranged from 1.79% to 2.20% at 15 days following the last dose and for the single cohort given sabirnetug Q2W, the value was 1.65% at seven days following the last dose. Because this is a ratio of total to free sabirnetug, these values cannot be readily compared to values of other monoclonal antibodies. For both single-dose and multiple-dose sabirnetug administration, there was a modest correlation between CSF sabirnetug concentrations and serum sabirnetug concentrations, and CSF sabirnetug concentrations appeared to increase in a dose-related manner.

### 3.8 Central target engagement of sabirnetug in cerebrospinal fluid

**Fig. 3:**
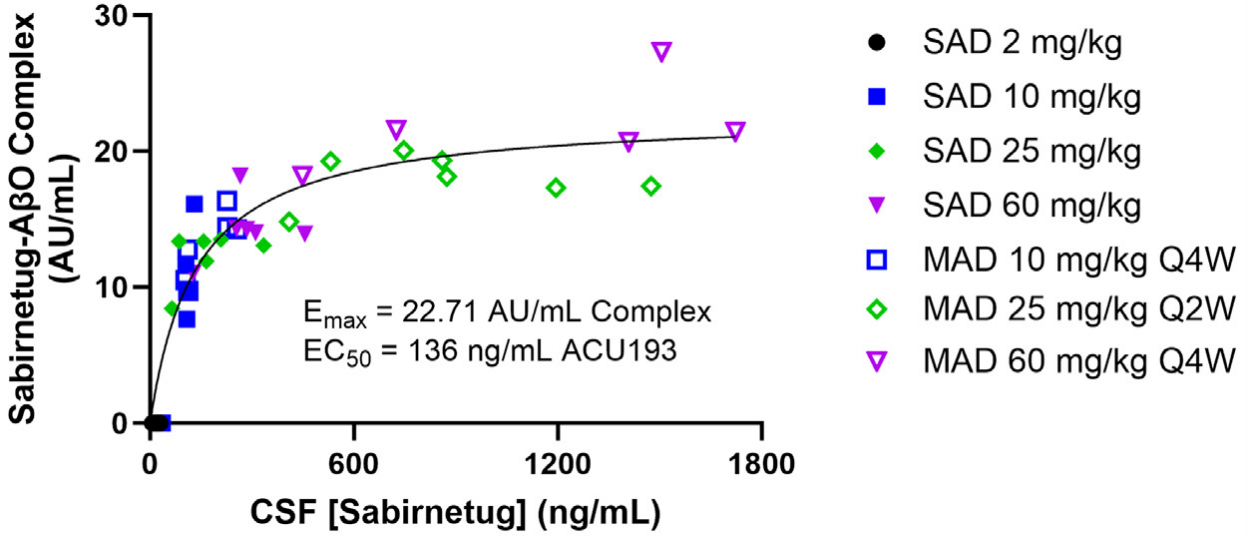
Exposure-Response Relationship between Sabirnetug-AβO Complex and Sabirnetug Concentrations in CSF with E_max_ Model (Pharmacokinetics Population). AU, arbitrary units; E_max_, maximum exposure; EC_50_, effective concentration 50% (half-maximal effective concentration of sabirnetug); MAD, multiple ascending dose (study Part B); AβO, amyloid β oligomers; SAD, single ascending dose (study Part A).

CSF target engagement (i.e., sabirnetug-AβO complex) was dose and exposure dependent in cohorts 2 through 7 (Fig. 3). Pharmacokinetic/pharmacodynamic modeling of the relationship between target engagement and sabirnetug concentration in CSF shows that target engagement approached maximal response in cohorts 6 (60 mg/kg Q4W) and 7 (25 mg/kg Q2W; E_max_ = 22.71 AU/mL sabirnetug-AβO complex. The model shows that the concentrations of sabirnetug administered in these cohorts approached saturation of AβO binding in the CSF of these participants. No correlation with target engagement was observed for *APOE* ε4 genotype or presence of ARIA, or for baseline amyloid burden (data not shown).

### 3.9 Positron emission tomography

Pooled values for participants in cohorts 1, 2, 3, and 4 who received placebo were 48.5 Centiloids at baseline and 49.5 Centiloids at day 42 (change from baseline, +1.0 Centiloids, +2.2%). Participants in cohorts 1, 2, 3, and 4 who received sabirnetug had mean baseline Centiloid values of 65.3, 70.0, 63.7, and 60.2, respectively, and mean values at day 42 of 62.7 Centiloids (mean change from baseline, −2.6 Centiloids, −4.0%), 84.9 Centiloids (mean change, +14.9 Centiloids, +21.4%), 62.9 (mean change, −0.8 Centiloids, −1.3%), and 50.3 (mean change, −9.9 Centiloids, −16.5%), respectively.

The pooled value for participants in cohorts 5 and 6 who received placebo was 37.3 Centiloids at baseline. At day 70, mean value for cohort 5 was 44.6 Centiloids (change from baseline, 7.3 Centiloids, 19.5%). At day 63, mean value for cohort 6 was 28.6 Centiloids (mean change, −8.7 Centiloids, −23.2%). For sabirnetug-treated participants in cohort 5, mean baseline value was 55.8 Centiloids and day 70 value was 59.2 Centiloids (mean change from baseline, +3.4 Centiloids, +6.1%, *p*=0.746). For sabirnetug-treated participants in cohort 6, baseline value was 71.9 Centiloids and day 63 value was 53.7 Centiloids (mean change, −18.2 Centiloids, −23.2%; *p*=0.01). For sabirnetug-treated participants in cohort 7, baseline value was 66.8 Centiloids and day 70 value was 53.0 Centiloids (mean change, −18.3 Centiloids, −25.6%; *p*=0.01).

### 3.10 Cognitive efficacy assessments

No clinically important mean changes from baseline to endpoint were noted for the exploratory clinical outcome measures ADAS-Cog13, CCTB, MMSE, CDR-SB, ADCS-ADL, or NPI (Supplemental Tables 1 and 2).

## 4 Discussion

In INTERCEPT-AD, a first-in-human study in participants with early symptomatic AD, sabirnetug, a monoclonal antibody highly selective for soluble globular AβOs [24], was well tolerated, demonstrating a relatively low occurrence of treatment-emergent ARIA-E or infusion reactions. Of 48 participants given sabirnetug, five (10%) developed ARIA-E, including one instance that was mildly symptomatic (2% overall frequency). Of the five participants with ARIA-E, one received three administrations of 10 mg/kg, one received three administrations of 25 mg/kg sabirnetug, and three received one or three administrations of 60 mg/kg sabirnetug. The single symptomatic participant had received a single infusion of 60 mg/kg sabirnetug and experienced mild leg dysfunction, which resolved within four weeks, as did the ARIA-E.

Infusion reactions/hypersensitivity were not frequent (6.3% for sabirnetug versus 0.0% for placebo) and were not severe. Treatment-emergent anti-sabirnetug antibodies, all at titers ranging from 1 to 8, were reported for six of 48 participants given sabirnetug.

Given that sabirnetug was developed to bind to soluble species of globular AβOs rather than to amyloid plaque, and based on prior nonclinical studies of sabirnetug or its murine precursor, the ability of sabirnetug to affect plaque load in humans was previously unknown. However, plaque reduction was observed over three months treatment in the MAD portion of the study. This represented approximate reductions of 25% and 20% Centiloids in the 60 mg/kg Q4W and 25 mg/kg Q2W cohorts, respectively. Although sabirnetug is an IgG2, it may have some phagocytic activity. Sabirnetug may be less selective for AβOs versus plaques in humans than in nonhuman primates, or sabirnetug may decrease plaque load as a result of shifts in equilibrium related to clearance of soluble globular AβOs. These three possibilities are not mutually exclusive.

Assessment of target engagement in CSF was an important component of the study, which, in combination with safety data, led to an informed choice of doses for the ongoing ALTITUDE-AD Phase 2 trial (NCT06335173) [[29]]. As shown in Fig. 3, concentrations of the sabirnetug-AΒO complex approached E_max_ for participants receiving 25 mg/kg Q2W (cohort 7) and 60 mg/kg Q4W (cohort 6). Additional modeling analyses will be reported separately. Target engagement modeling indicates that sabirnetug successfully bound AβOs in dosed participants and suggests that sabirnetug is either binding AβOs in the CSF and being cleared from the CSF or binding AβOs in both the brain and the CSF and being cleared from both. Clearance of AβOs from the CSF should also result in lower concentrations in the brain as concentrations in the brain and CSF are in equilibrium.

Based on the target engagement data and safety data from this INTERCEPT-AD study, selected doses for the ALTITUDE-AD study are 35 mg/kg Q4W and 50 mg/kg Q4W. Modeling based on participants in the 50^th^ percentile of target engagement predicted that, with a dose of 35 mg/kg Q4W, 85.1% of maximum target engagement would occur at peak sabirnetug concentration and 71.1% of maximum target engagement is predicted at trough. With a dose of 50 mg/kg Q4W, peak and trough target engagement would be 89.1% and 77.9% of maximum target engagement, respectively [][30]. A dose of 50 mg/kg is also likely to provide plaque reduction based on results from INTERCEPT-AD. Thus, the 18-month ALTITUDE-AD study of sabirnetug was designed as a three-arm study using 35 mg/kg Q4W and 50 mg/kg Q4W sabirnetug and placebo.

As expected in a Phase 1 study with a maximum of three administrations per participant, no statistically significant changes in clinical measures including the ADAS-Cog13, CCTB, MMSE, CDR-SB, ADCS-ADL, or NPI were seen. However, the data indicate central target engagement and amyloid plaque reduction consistent with an effect of sabirnetug on the AD pathologic process after only three administrations. CSF and plasma biomarker results from the INTERCEPT-AD trial have been presented [31] and will be published in a subsequent manuscript.

Taken together, the INTERCEPT-AD Phase 1 study of sabirnetug in participants with early symptomatic AD provided information that aided in the design of the subsequent Phase 2 study. Although the decrease in amyloid plaque was not anticipated, these preliminary findings suggest sabirnetug may facilitate simultaneous clearance of multiple forms of Aβ. Sabirnetug was well-tolerated and a novel target engagement assay provided data that along with safety data informed the selection of doses for the Phase 2 ALTITUDE-AD study.

## Funding

This study was funded by Acumen Pharmaceuticals, Inc.

*Role of the sponsor:* Employees of Acumen Pharmaceuticals designed and conducted the study, in collaboration with investigators, including data collection and analysis. Employees of Acumen Pharmaceuticals participated as authors on this paper and drafted sections of the manuscript. Employees of Acumen Pharmaceuticals also reviewed the manuscript and provided comments for consideration by the authors.

## Declaration of competing interest

None.

## Appendix Supplementary materials

Image, application 1
