# Supplementary material for: INTERCEPT-AD, a phase 1 study of intravenous sabirnetug in participants with mild cognitive impairment or mild dementia due to Alzheimer's disease
Source: J Prev Alzheimers Dis. 2025 Jan 1;12(1):100005. doi: 10.1016/j.tjpad.2024.100005 (PMC12184067; doi:10.1016/j.tjpad.2024.100005)
Supplement: Supplementary file 1 [file mmc1.docx]

**INTERCEPT-AD, A Phase 1 Study of Intravenous Sabirnetug in Participants with Mild Cognitive Impairment or Mild Dementia Due to Alzheimer’s Disease**

**Eric Siemers, Todd Feaster, Gopalan Sethuraman, Karen Sundell, Vladimir Skljarevski, Erika N. Cline, Hao Zhang, Jasna Jerecic, Lawrence S. Honig, Stephen Salloway, Reisa Sperling, Mirjam N. Trame, Michael G. Dodds, and Kimball Johnson**

**Supplemental Materials**

**Supplemental Table 1. Cognitive Assessments in Study Part A (Single Ascending Dose)**

|  | **Baseline** | **Day 2** | **Day 3** | **Day 4** | **Day 7** | **Day 21** |
| --- | --- | --- | --- | --- | --- | --- |
| **Alzheimer’s Disease Assessment Scale – Cognitive Subscale, 13 Item (ADAS-Cog13)** | | | | | | |
| Cohort 1 (N=6) | 21.3 (13.59) | - | - | - | - | 23.5 (9.27) |
| Cohort 2 (N=6) | 27.0 (6.87) | - | - | - | - | 26.8 (4.96) |
| Cohort 3 (N=6) | 23.0 (9.36) | - | - | - | - | 20.2 (8.06) |
| Cohort 4 (N=6) | 25.3 (6.89) | - | - | - | - | 19.2 (5.15) |
| Pooled Placebo (N=8) | 24.9 (3.68) | - | - | - | - | 20.4 (5.80) |
| **Computerized Cognitive Testing Battery – Cognition Composite Score (CCTB-CCS)*** | | | | | | |
| Cohort 1 (N=6) | 0.42 (0.703) | 0.63 (0.556) | 0.60 (0.562) | 0.69 (0.428) | 0.67 (0.468) | 0.83 (0.343) |
| Cohort 2 (N=6) | -0.32 (0.474) | -0.56 (0.600) | -0.11 (0.640) | -0.18 (0.530) | -0.16 (0.631) | -0.34 (0.658) |
| Cohort 3 (N=6) | 0.20 (0.547) | 0.11 (0.385) | 0.08 (0.368) | 0.50 (0.339) | 0.40 (0.594) | 0.38 (0.416) |
| Cohort 4 (N=6) | 0.03 (0.759) | -0.10 (0.236) | -0.02 (0.556) | 0.04 (0.652) | 0.17 (0.513) | 0.20 (0.739) |
| Pooled Placebo (N=8) | 0.14 (0.410) | 0.07 (0.510) | -0.06 (0.638) | 0.26 (0.338) | 0.51 (0.551) | 0.41 (0.532) |
| **Mini-Mental State Examination (MMSE) Total Score^†^** | | | | | | |
| Cohort 1 (N=6) | 25.5 (3.67) | - | - | - | - | 27.2 (2.93) |
| Cohort 2 (N=6) | 24.0 (2.61) | - | - | - | - | 21.5 (2.74) |
| Cohort 3 (N=6) | 24.2 (4.49) | - | - | - | - | 25.2 (2.79) |
| Cohort 4 (N=6) | 25.5 (2.43) | - | - | - | - | 24.5 (3.89) |
| Pooled Placebo (N=8) | 24.1 (3.27) | - | - | - | - | 25.0 (2.78) |
| **Clinical Dementia Rating – Sum of Boxes (CDR-SB)** | | | | | | |
| Cohort 1 (N=6) | 3.42 (1.908) | - | - | - | - | 3.08 (2.084) |
| Cohort 2 (N=6) | 4.00 (1.761) | - | - | - | - | 3.75 (1.405) |
| Cohort 3 (N=6) | 3.75 (1.605) | - | - | - | - | 3.08 (1.563) |
| Cohort 4 (N=6) | 3.75 (1.214) | - | - | - | - | 3.75 (0.418) |
| Pooled Placebo (N=8) | 3.38 (1.885) | - | - | - | - | 2.69 (1.335) |
| **Alzheimer’s Disease Cooperative Study – Activities of Daily Living (ADCS-ADL) Total Score** | | | | | | |
| Cohort 1 (N=6) | 73.3 (3.14) | - | - | - | - | 70.0 (10.79) |
| Cohort 2 (N=6) | 65.8 (14.95) | - | - | - | - | 67.0 (16.80) |
| Cohort 3 (N=6) | 71.8 (3.49) | - | - | - | - | 71.3 (5.20) |
| Cohort 4 (N=6) | 72.2 (3.87) | - | - | - | - | 71.3 (3.56) |
| Pooled Placebo (N=8) | 71.8 (5.06) | - | - | - | - | 72.6 (3.25) |
| **Neuropsychiatric Inventory (NPI) Total Score** | | | | | | |
| Cohort 1 (N=6) | 4.0 (3.16) | - | - | - | - | 1.8 (2.40) |
| Cohort 2 (N=6) | 10.3 (16.66) | - | - | - | - | 7.8 (11.16) |
| Cohort 3 (N=6) | 4.2 (3.71) | - | - | - | - | 3.3 (3.93) |
| Cohort 4 (N=6) | 4.5 (4.72) | - | - | - | - | 5.0 (4.77) |
| Pooled Placebo (N=8) | 6.9 (8.61) | - | - | - | - | 4.4 (9.36) |
| **Integrated Alzheimer’s Disease Rating Scale** | | | | | | |
| Cohort 1 (N=6) | 118.3 (15.21) | - | - | - | - | 112.8 (11.50) |
| Cohort 2 (N=6) | 105.8 (14.44) | - | - | - | - | 106.7 (18.37) |
| Cohort 3 (N=6) | 115 (12.51) | - | - | - | - | 117.3 (9.40) |
| Cohort 4 (N=6) | 112.8 (9.81) | - | - | - | - | 118.5 (7.29) |
| Pooled Placebo (N=8) | 113.1 (5.28) | - | - | - | - | 118.4 (7.98) |

Reported values are mean (standard deviation). Dashes indicate timepoints when the assessment was not performed.

*The CCTB-Composite Score included the following subtests: the Cogstate Brief Battery (detection test, identification test, one card learning, and one card back), the International Daily Symbol Substitution Test, the International Shopping List Test (immediate and delayed recall), and the Modified Groton Maze Learning Test. CCTB-Composite Score was a z-score used where higher values indicate better performance on the test.

^†^Initial Mini-Mental State Examination was performed at screening rather than baseline.

**Supplemental Table 2. Cognitive Assessments in Study Part B (Multiple Ascending Dose)**

| **Cohort 5, 10 mg/kg Sabirnetug Q4W (N=8)** | | | | | | | | | | | | | | | | |
| --- | --- | --- | --- | --- | --- | --- | --- | --- | --- | --- | --- | --- | --- | --- | --- | --- |
|  | **Baseline** | | **Day 7** | | | **Day 28** | | | **Day 56** | | | **Day 70** | | | **Day 196** | |
| **ADAS-Cog13** | 28.0 (12.15) | | - | | | - | | | - | | | 28.6 (7.46) | | | 27.7 (12.41) | |
| **CCTB-CCS*** | -0.31 (0.909) | | -0.7 (0.620) | | | -0.17 (0.868) | | | 0.08 (0.822) | | | -0.11 (0.623) | | | 0.28 (0.581) | |
| **MMSE^†^** | 23.8 (3.65) | | - | | | - | | | - | | | 22.4 (3.46) | | | 22.1 (5.37) | |
| **CDR-SB** | 4.19 (1.751) | | - | | | - | | | - | | | 4.13 (1.847) | | | 6.43 (3.611) | |
| **ADCS-ADL** | 65.5 (5.01) | | - | | | - | | | - | | | 63.6 (8.77) | | | 60.4 (12.20) | |
| **NPI** | 2.6 (2.51) | | - | | | - | | | - | | | 3.8 (4.17) | | | - | |
| **iADRS** | 104.0 (14.86) | | - | | | - | | | - | | | 101.8 (14.29) | | | 99.6 (20.84) | |
| **Cohort 6, 60 mg/kg Sabirnetug Q4W (N=8)** | | | | | | | | | | | | | | | | |
|  | **Baseline** | | **Day 7** | | | **Day 28** | | | **Day 56** | | | **Day 63** | | | **Day 126** | |
| **ADAS-Cog13** | 23.7 (8.28) | | - | | | - | | | - | | | 23.4 (6.07) | | | 27.8 (9.85) | |
| **CCTB-CCS** | 0.08 (0.730) | | -0.07 (0.781) | | | 0.14 (0.731) | | | 0.29 (0.638) | | | -0.4 (0.702) | | | -0.14 (0.820) | |
| **MMSE^†^** | 23.0 (4.41) | | - | | | - | | | - | | | 23.5 (44.4) | | | 23.6 (4.53) | |
| **CDR-SB** | 2.88 (1.217) | | - | | | - | | | - | | | 3.25 (1.035) | | | 3.00 (1.069) | |
| **ADCS-ADL** | 69.4 (8.62) | | - | | | - | | | - | | | 69.9 (7.81) | | | - | |
| **NPI** | 3.0 (3.96) | | - | | | - | | | - | | | 1.5 (1.77) | | | - | |
| **iADRS** | 112.0 (14.58) | | - | | | - | | | - | | | 113.1 (9.89) | | | 109.1 (14.72) | |
| **Q4W Placebo**^‡^ **(N=4 for baseline and Days 7, 28, and 56, N=2 for Days 63, 70, 126, and 196)** | | | | | | | | | | | | | | | | |
|  | **Baseline** | **Day 7** | | | **Day 28** | | **Day 56** | | **Day 63** | | **Day 70** | | **Day 126** | | | **Day 196** |
| **ADAS-Cog13** | 21.5 (13.92) | - | | | - | | - | | 32.0 (15.56) | | 18.0 (5.66) | | 34.5 (13.44) | | | 11.0 (1.41) |
| **CCTB-CCS** | -0.21 (0.510) | -0.27 (0.851) | | | 0.03 (0.504) | | -0.02 (0.951) | | -0.20 (0.918) | | 0.30 (0.397) | | -0.44 (0.935) | | | -0.09 (0.544) |
| **MMSE^†^** | 26.0 (4.83) | - | | | - | | - | | 21.5 (4.95) | | 28.0 (0.00) | | 21.5 (4.95) | | | 26.0 (1.41) |
| **CDR-SB** | 3.50 (2.121) | - | | | - | | - | | 3.75 (1.786) | | 3.50 (0.707) | | 4.75 (1.061) | | | 5.00^§^ |
| **ADCS-ADL** | 59.3 (14.31) | - | | | - | | - | | 58.5 (9.19) | | 61.5 (3.54) | | - | | | 60.0^§^ |
| **NPI** | 17.5 (22.05) | - | | | - | | - | | 10.5 (7.78) | | 3.0 (4.24) | | - | | | - |
| **iADRS** | 104.8 (20.09) | - | | | - | | - | | 93.0 (5.66) | | 109.5 (9.19) | | 91.5 (0.71) | | | 114.0 |
| **Cohort 7, 25 mg/kg Sabirnetug Q2W (N=8)** | | | | | | | | | | | | | | | | |
|  | **Baseline** | | | **Day 7** | | | | **Day 28** | | **Day 35** | | | | **Day 98** | | |
| **ADAS-Cog13** | 24.6 (9.68) | | | - | | | | - | | 22.4 (8.19) | | | | 20.5 (10.82) | | |
| **CCTB-CCS** | 0.30 (0.781) | | | 0.32 (0.782) | | | | 0.41 (0.780) | | 0.50 (0.734) | | | | 0.51 (0.711) | | |
| **MMSE^†^** | 24.3 (4.03) | | | - | | | | - | | 24.3 (4.50) | | | | 24.0 (5.40) | | |
| **CDR-SB** | 3.56 (3.145) | | | - | | | | - | | 3.44 (2.665) | | | | 3.94 (3.087) | | |
| **ADCS-ADL** | 71.1 (7.56) | | | - | | | | - | | 71.6 (5.88) | | | | - | | |
| **NPI** | 3.7 (8.98) | | | - | | | | - | | 4.4 (8.31) | | | | - | | |
| **iADRS** | 112.4 (12.83) | | | - | | | | - | | 115.4 (10.80) | | | | 116.3 (14.10) | | |
| **Placebo Q2W (N=2)** | | | | | | | | | | | | | | | | |
|  | **Baseline** | | | **Day 7** | | | | - | | **Day 35** | | | | **Day 98** | | |
| **ADAS-Cog13** | 24.5 (14.85) | | | - | | | | - | | 20.5 (10.61) | | | | 21.0 (16.97) | | |
| **CCTB-CCS** | 0.07 (0.750) | | | -0.17 (0.598) | | | | -0.25 (0.507) | | 0.11 (0.861) | | | | 0.14 (0.766) | | |
| **MMSE^†^** | 25.0 (4.24) | | | - | | | | - | | 26.5 (4.95) | | | | 26.5 (4.95) | | |
| **CDR-SB** | 1.75 (0.354) | | | - | | | | - | | 1.50 (0.707) | | | | 2.00 (1.414) | | |
| **ADCS-ADL** | 72.5 (3.54) | | | - | | | | - | | 72.0 (7.07) | | | | - | | |
| **NPI** | 1.0 (1.41) | | | - | | | | - | | 1.0 (1.41) | | | | - | | |
| **iADRS** | 114.0 (18.38) | | | - | | | | - | | 117.5 (17.68) | | | | 116.0 (24.04) | | |

ADAS-Cog13, Alzheimer’s Disease Assessment Scale – Cognitive Subscale, 13 Item. ADCS-ADL, Alzheimer’s Disease Cooperative Study – Activities of Daily Living (Total Score). CCTB-CCS, Computerized Cognitive Testing Battery – Cognition Composite Score. CDR-SB, Clinical Dementia Rating – Sum of Boxes. iADRS, Integrated Alzheimer’s Disease Rating Scale; MMSE, Mini-Mental State Examination (Total Score). NPI, Neuropsychiatric Inventory (Total Score). SD, Standard Deviation.

*The CCTB-Composite Score included the following subtests:  the Cogstate Brief Battery (detection test, identification test, one card learning, and one card back), the International Daily Symbol Substitution Test, the International Shopping List Test (immediate and delayed recall), and the Modified Groton Maze Learning Test. CCTB-Composite Score was a z-score used where higher values indicate better performance on the test.

^†^Initial Mini-Mental State Examination was performed at screening rather than baseline.

^‡^Pooled results from participants who received placebo in Cohorts 5 and 6.

^§^N=1 at this timepoint; SD could not be calculated

Values indicate mean (SD). Dashes indicate timepoints when the assessment was not performed.
